# Safety, efficacy, and survival outcomes of immune checkpoint inhibitors rechallenge in patients with cancer: a systematic review and meta-analysis

**DOI:** 10.1093/oncolo/oyae134

**Published:** 2024-06-28

**Authors:** Shi-Jia Liu, Lun-Jie Yan, Han-Chao Wang, Zi-Niu Ding, Hui Liu, Xiao Zhang, Guo-Qiang Pan, Cheng-Long Han, Bao-Wen Tian, Xiao-Rong Yang, Si-Yu Tan, Zhao-Ru Dong, Dong-Xu Wang, Yu-Chuan Yan, Tao Li

**Affiliations:** Department of General Surgery, Qilu Hospital of Shandong University, Jinan 250012, People’s Republic of China; Department of General Surgery, Qilu Hospital of Shandong University, Jinan 250012, People’s Republic of China; Institute for Financial Studies, Shandong University, Jinan 250100, People’s Republic of China; Department of General Surgery, Qilu Hospital of Shandong University, Jinan 250012, People’s Republic of China; Department of General Surgery, Qilu Hospital of Shandong University, Jinan 250012, People’s Republic of China; Department of General Surgery, Qilu Hospital of Shandong University, Jinan 250012, People’s Republic of China; Department of General Surgery, Qilu Hospital of Shandong University, Jinan 250012, People’s Republic of China; Department of General Surgery, Qilu Hospital of Shandong University, Jinan 250012, People’s Republic of China; Department of General Surgery, Qilu Hospital of Shandong University, Jinan 250012, People’s Republic of China; Clinical Epidemiology Unit, Qilu Hospital of Shandong University, Jinan 250012, People’s Republic of China; Department of General Surgery, Qilu Hospital of Shandong University, Jinan 250012, People’s Republic of China; Department of General Surgery, Qilu Hospital of Shandong University, Jinan 250012, People’s Republic of China; Department of General Surgery, Qilu Hospital of Shandong University, Jinan 250012, People’s Republic of China; Department of General Surgery, Qilu Hospital of Shandong University, Jinan 250012, People’s Republic of China; Department of General Surgery, Qilu Hospital of Shandong University, Jinan 250012, People’s Republic of China

**Keywords:** immune checkpoint inhibitors, immune-related adverse events, rechallenge, neoplasms, survival analysis, meta-analysis

## Abstract

**Backgrounds:**

There is little evidence on the safety, efficacy, and survival benefit of restarting immune checkpoint inhibitors (ICI) in patients with cancer after discontinuation due to immune-related adverse events (irAEs) or progressive disease (PD). Here, we performed a meta-analysis to elucidate the possible benefits of ICI rechallenge in patients with cancer.

**Methods:**

Systematic searches were conducted using PubMed, Embase, and Cochrane Library databases. The objective response rate (ORR), disease control rate (DCR), progression-free survival (PFS), overall survival (OS), and incidence of irAEs were the outcomes of interest.

**Results:**

Thirty-six studies involving 2026 patients were analyzed. ICI rechallenge was associated with a lower incidence of all-grade (OR, 0.05; 95%CI, 0.02-0.13, *P* < .05) and high-grade irAEs (OR, 0.37; 95%CI, 0.21-0.64, *P* < .05) when compared with initial ICI treatment. Though no significant difference was observed between rechallenge and initial treatment regarding ORR (OR, 0.69; 95%CI, 0.39-1.20, *P* = .29) and DCR (OR, 0.85; 95%CI, 0.51-1.40, *P* = 0.52), patients receiving rechallenge had improved PFS (HR, 0.56; 95%CI, 0.43-0.73, *P* < .05) and OS (HR, 0.55; 95%CI, 0.43-0.72, *P* < .05) than those who discontinued ICI therapy permanently. Subgroup analysis revealed that for patients who stopped initial ICI treatment because of irAEs, rechallenge showed similar safety and efficacy with initial treatment, while for patients who discontinued ICI treatment due to PD, rechallenge caused a significant increase in the incidence of high-grade irAEs (OR, 4.97; 95%CI, 1.98-12.5, *P* < .05) and a decrease in ORR (OR, 0.48; 95%CI, 0.24-0.95, *P* < .05).

**Conclusion:**

ICI rechallenge is generally an active and feasible strategy that is associated with relative safety, similar efficacy, and improved survival outcomes. Rechallenge should be considered individually with circumspection, and randomized controlled trials are required to confirm these findings.

## Teaser

Whether to resume immune checkpoint inhibitor therapy (ICI) after discontinuation is controversial because of the unclear safety, efficacy, and survival benefits of the rechallenge. This article reports the possible benefits of ICI rechallenge in patients with cancer.

## Graphical Abstract

**Graphical Abstract:**
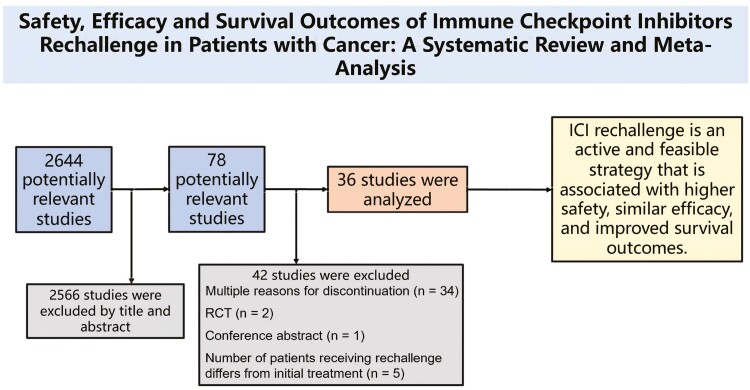

**Implications for practice**

Whether to resume ICI therapy after discontinuation remains controversial in the real-world practice, because the safety, efficacy, and long-term survival of the rechallenge have yet to be clarified. Our study found that the incidence of all-grade and high-grade irAEs was lower in patients after rechallenge when compared with initial ICI therapy. Though there was no significant difference in efficacy, resuming ICI treatment provided better survival outcomes. Thus, rechallenge can be considered individually.

## Introduction

The development of immune checkpoint inhibitors (ICI) targeting cytotoxic T lymphocyte antigen 4 (CTLA-4), programmed cell death protein 1 (PD-1), or their ligands (PD-L1) is a milestone in cancer therapy,^1^ and growing evidence suggests that they induce durable treatment responses and prolong survival across multiple types of cancer.^2-4^ Recently, the clinical application of anti-lymphocyte activation gene 3 (LAG-3) therapy has been approved by FDA, providing new options for cancer patients.^5^ Nevertheless, many patients may eventually discontinue ICI therapy because of disease progression. Even in patients with favorable therapeutic efficacy,^6^ ICI treatment may not last long because of severe toxicities.^7^ Toxicity due to immune-related adverse events (irAEs) has gradually become one of the most important reasons impeding the long-term clinical benefits in patients with cancer. Whether to resume ICI therapy after discontinuation remains controversial because the safety, efficacy, and long-term survival of the rechallenge have yet to be clarified.

Based on the latest NCCN guidelines,^8^ permanent discontinuation of a given class of immunotherapy is typically warranted in the setting of severe irAEs induced by that class of immunotherapy and may be warranted in the setting of moderate irAEs, while resumption of immunotherapy following grade 2 irAEs can be considered. Experts from SITC,^9^ ESMO,^10^ and ASCO^11^ also advise exercising caution when reintroducing ICI treatment to patients who previously suffered grade 3-4 irAEs due to the potential risk of severe toxicity resurfacing. Nevertheless, these recommendations are mainly based on expert consensus and need to be verified using more high-quality evidence.

From 2020 to 2023, several meta-analyses^12-17^ synthesized the evidence, but no consistent conclusion has been reached on whether ICI rechallenge is a preferable option. The previously reported results were generally extracted from studies with heterogeneous populations, leading to difficulty in interpreting the results. Furthermore, previous analyses included only a small number of studies with multiple reasons for discontinuing ICI treatment, did not compare rechallenge outcomes with those of initial ICI treatment and lacked detailed subgroup analysis. New strategies are needed to overcome these limitations in future studies.

In this study, we critically assessed the existing literature in this updated systematic review and meta-analysis, which included several newly published articles and strictly specified the reasons for suspending initial ICI treatment. We also explored the sources of heterogeneity using predefined subgroup and meta-regression analyses. Notably, we also compared the survival outcomes between patients resuming ICI therapy and permanently ceasing ICI treatment.

## Methods

We followed the guidelines outlined in the Preferred Reporting Items for Systematic Review and Meta-Analyses (PRISMA) to report this systematic review. This systematic review was prospectively registered at PROSPERO (https://www.crd.york.ac.uk/prospero) as CRD42023409943.

### Search strategy

Two authors (Shi-Jia Liu and Lun-Jie Yan) conducted a comprehensive systematic search of articles using PubMed, EMBASE, Web of Science, and Cochrane Library databases in January 2023, following the PRISMA guidelines. Any disagreements were resolved by discussion and consensus. The main keywords used for search included cancer, tumor, neoplasm, immune checkpoint inhibitors (anti-PD-1, anti-PD-L1, anti-CTLA-4, anti-LAG-3, ICI), immunotherapies, specific ICI names (nivolumab, pembrolizumab, atezolizumab, durvalumab, avelumab, ipilimumab, tremelimumab, cemiplimab, dostarlimab and relatlimab), and terms relevant to “rechallenge” (retreat, readministrate, restart, reinitiate, resume, reintroduction, retreatment, reintroduction, reinduction).

### Inclusion and exclusion criteria

Full-text articles in English were included in the database search. The inclusion criteria were as follows: (1) enrolled adult patients with solid tumors, (2) patients treated with ICI, (3) patients who resumed treatment after a previous interruption due to toxicity or progression, and (4) reported outcomes including toxicity, efficacy, PFS, or OS. The exclusion criteria were as follows: (1) patients who stopped ICI therapy for other reasons; (2) no detailed information on irAEs or treatment outcomes of ICI; (3) non-clinical studies; (4) reviews and meta-analyses; (5) case reports, editorials, and letters to the editors; (6) studies with insufficient data or no information available; and (7) animal experiments. Articles that met the inclusion criteria were retrieved for full-text evaluation.

### Data extraction and quality assessment

The required data from the eligible studies were independently extracted and recorded by 2 independent researchers using a predefined electronic spreadsheet, and all discrepancies were resolved by consensus. The following detailed characteristics of the study were extracted: author, publication year, study design, cancer type, whether combined treatments (such as radiotherapy, chemotherapy, or targeted therapy) were administered during treatment; type of initial and rechallenged ICI; cessation reason of initial ICI treatment; incidence of irAEs after the initial and rechallenge treatment; ORR and DCR after initial or rechallenge therapy; HR of PFS and OS in patients received rechallenge or permanently discontinued ICI treatment. Considering that the included studies were all retrospective, the same 2 independent researchers performed methodological quality assessments according to the Newcastle-Ottawa Scale (NOS), which evaluates the study design based on 8 questions regarding population selection, comparability, and exposure.

### Outcomes

The safety assessment included the incidence of all-grade and high-grade irAEs. The severity of irAEs was recorded as grades 1 to 5 according to version 4 or 5 of the Common Terminology Criteria for Adverse Events (CTCAE) of the National Cancer Institute (Bethesda, MD, USA). Grade ≥ 3 was considered as high-grade irAE, whereas grades 1 or 2 were low-grade irAEs. The efficacy assessments included ORR and DCR. ORR was defined as the proportion of patients with a complete or partial response (CR or PR), whereas DCR was defined as the proportion of patients with a complete or partial response or stable disease (SD), according to the Response Evaluation Criteria in Solid Tumors version 1.1 (RECIST 1.1). Of note, Alaiwi et al,^18^ Awidi et al,^19^ and Ravi et al^20^ provided the outcomes of patients rechallenged with the same or different ICI. Therefore, each of the studies was considered as 2 independent studies to be included in the analysis.

### Data analysis

Stata/SE (version 15.1) was used for statistical analyses and plotting. Synthesis of all-grade and high-grade irAEs, ORR, and DCR was performed using the pooled odds ratio (OR) with 95% CI. Because the studies included in the meta-analysis were all retrospective, the random effects model was adopted, considering the possible significant heterogeneity. HR and 95% CI for OS and PFS were extracted directly based on data from univariate or, preferentially, if possible, multivariable Cox proportional hazard models. Between-study heterogeneity was calculated using Higgins *I*^2^ statistics. *I*^2^ < 50% was considered to indicate low heterogeneity, and the fixed-effect model was adopted. Individual effects and pooled mean effect sizes were summarized in forest plots for each outcome. A sensitivity analysis was conducted to assess the extent to which each study contributed to the results and heterogeneity and to confirm the robustness of the primary analysis results.

Predefined subgroup analysis was conducted for covariates that may have contributed to the heterogeneity of the pooled results, including the reason for discontinuation, whether rechallenged with the same ICI, tumor type, and whether received combined treatments (such as radiotherapy, chemotherapy, or targeted therapy) during ICI treatment. Meta-regression analysis was performed with the above covariates to determine between-study heterogeneity and the impact of covariates on the pooled estimates. Publication bias was visualized using funnel plots, and funnel plot asymmetry was assessed using Begg’s test. All tests were 2-sided, and *P*-value less than .05 was considered statistically significant.

## Results

### Study selection process and characteristics of included studies

**Figure 1.**
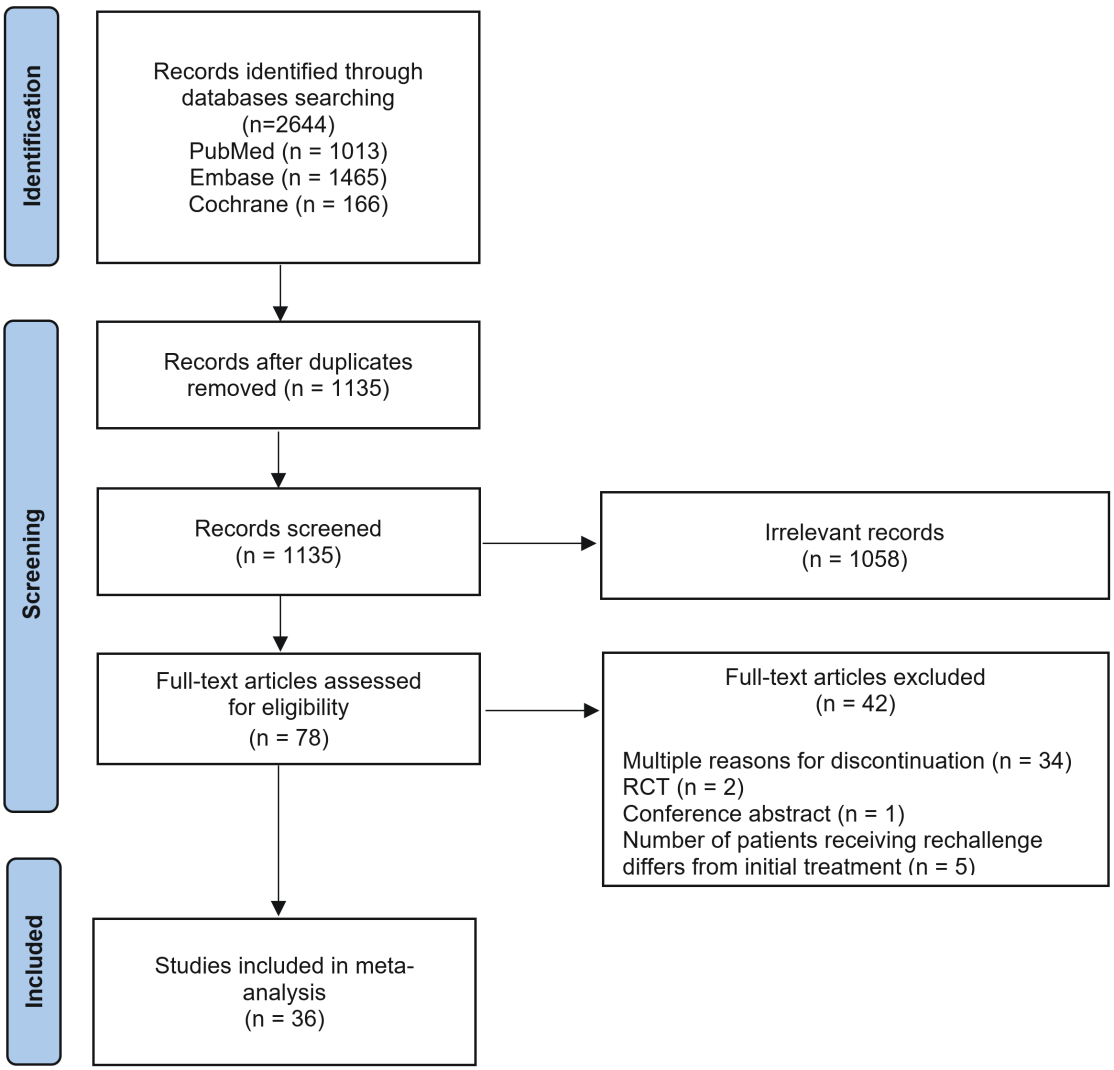
Flow diagram of the studies included in the meta-analysis.

Our literature search yielded 2644 studies. After removing duplicates and screening the titles and abstracts, 78 studies were retrieved for evaluation. In total, 36 studies comprising 2026 individuals with advanced cancer were included in the present study (Figure 1). The baseline characteristics of studies included for safety and efficacy analysis were summarized in Table 1 and Supplementary Table S1, and characteristics of studies included for survival analysis are summarized in Table 2. This study included patients with various cancers including non-small cell lung cancer (NSCLC), melanoma, renal cell carcinoma (RCC), colon cancer, and lymphoma. The initial ICI treatment included anti-PD-1/PD-L1 monotherapy (27/39, 69.2%), anti-CTLA-4 monotherapy (3/39, 7.7%), and dual immunotherapy (including 2 types of ICI) (9/39, 23.1%). The rechallenge treatment included anti-PD-1/PD-L1 monotherapy (26/39, 66.7%), anti-CTLA-4 monotherapy (2/39, 5.1%), and dual immunotherapy (11/39, 28.2%). Twenty-one (53.8%) studies used the same ICI for rechallenge. Sixteen (41%) studies included patients who had received combined treatments during ICI treatment. In 7 (17.9%) studies, the outcome was analyzed, and the HR of PFS and OS were provided.

**Table 1.** Characteristics of studies included for safety and efficacy analysis.

Cancer type			Melanoma	NSCLC	RCC	Multiple
Cause of interruption	irAEs		6/10	6/15	2/6	6/8
	PD		3/10	6/15	1/6	2/8
	irAEs/PD		1/10	3/15	3/6	0/8
Initial treatment	ICI type	PD-(L)1	5/10	15/15	4/6	3/8
		CTLA-4	3/10	0/15	0/6	5/8
		Dual	2/10	0/15	2/6	0/8
	All-grade irAEs		268/275	329/357	100/150	514/527
	High-grade irAEs		216/315	48/331	38/150	192/527
	ORR		41/218	207/503	59/285	9/40
	DCR		90/218	357/465	147/285	26/40
Rechallenge	ICI type	PD-(L)1	6/10	14/15	3/6	3/8
		CTLA-4	2/10	0/15	0/6	0/8
		Dual	2/10	1/15	3/6	5/8
	All-grade irAEs		133/275	137/357	78/150	210/527
	High-grade irAEs		79/315	27/331	24/150	62/527
	ORR		54/218	109/503	64/285	13/40
	DCR		96/218	337/465	164/285	28/40
Combined treatments	Yes		3/10	6/15	5/6	2/8
	No		7/10	9/15	1/6	6/8

“Multiple” means the cohort enrolled more than one kind of cancer patients; “Dual” means PD-(L)1 and CTLA-4; “Combined treatments” includes surgery, radiotherapy, chemotherapy, or targeted therapy.

Abbreviations: CTLA-4, cytotoxic T lymphocyte antigen 4; DCR, disease control rate; ICI, immune checkpoint inhibitors; irAEs, immune-related adverse events; NA, not applicable; NSCLC, non-small cell lung cancer; ORR, objective responses rate; PD, progressive disease; PD-1, programmed cell death protein 1; PD-L1, programmed cell death ligand 1; RCC, renal cell carcinoma.

**Table 2.** Characteristics of studies included for survival analysis.

Author	Year	Cancer	Initial ICI type	Cause of interruption	Rechallenge ICI type	PFS^*^	OS^*^
						Rechallenge vs discontinuation	Rechallenge vs discontinuation
Naqash^21^	2020	NSCLC	PD-(L)1	irAEs	PD-(L)1	0.57 (0.35-0.94)	0.38 (0.23-0.62)
Santini^22^	2018	NSCLC	PD-(L)1	irAEs	PD-(L)1	0.56 (0.3-1.03)	0.45 (0.21-1)
Fujisaki^23^	2021	NSCLC	PD-(L)1	irAEs	PD-(L)1	0.46 (0.16-1.41)	0.15 (0.02-1.1)
Yang^24^	2022	NSCLC	PD-(L)1	irAEs/PD	PD-(L)1	0.48 (0.19-1.23)	0.29 (0.08-1.03)
Li^25^	2022	NSCLC	PD-(L)1	PD	PD-(L)1	0.4 (0.24-0.67)	0.55 (0.29-1.04)
Guo^26^	2022	NSCLC	PD-(L)1	irAEs	PD-(L)1	1.15 (0.58-2.22)	0.91 (0.47-1.75)
Albandar^27^	2021	Multiple	Dual	irAEs	Dual	NA	0.84 (0.49-1.42)

^*^Data are presented as HR and 95%CI.

“Multiple” means the cohort enrolled more than one kind of patients with cancer; “Dual” means PD-(L)1 and CTLA-4.

Abbreviations: HR, hazard ratio; ICI, immune checkpoint inhibitors; irAEs, immune-related adverse events; NA, not applicable; NSCLC, non-small cell lung cancer; OS, overall survival; PD, progressive disease; PD-1, programmed cell death protein 1; PD-L1, programmed cell death ligand 1; PFS, progression-free survival.

Among all patients, the most common type of irAEs were colitis, rashes, pneumonitis, hepatitis, arthralgia, or endocrine toxic effects. Twenty (51.3%) studies included patients who stopped initial ICI treatment owing to irAEs. Twelve (30.8%) studies included patients who stopped initial ICI treatment due to PD, while the remaining 7 studies (17.9%) included patients who stopped ICI treatment for the above 2 reasons.

### Safety and efficacy of ICI rechallenge in the entire cohort

**Figure 2.**
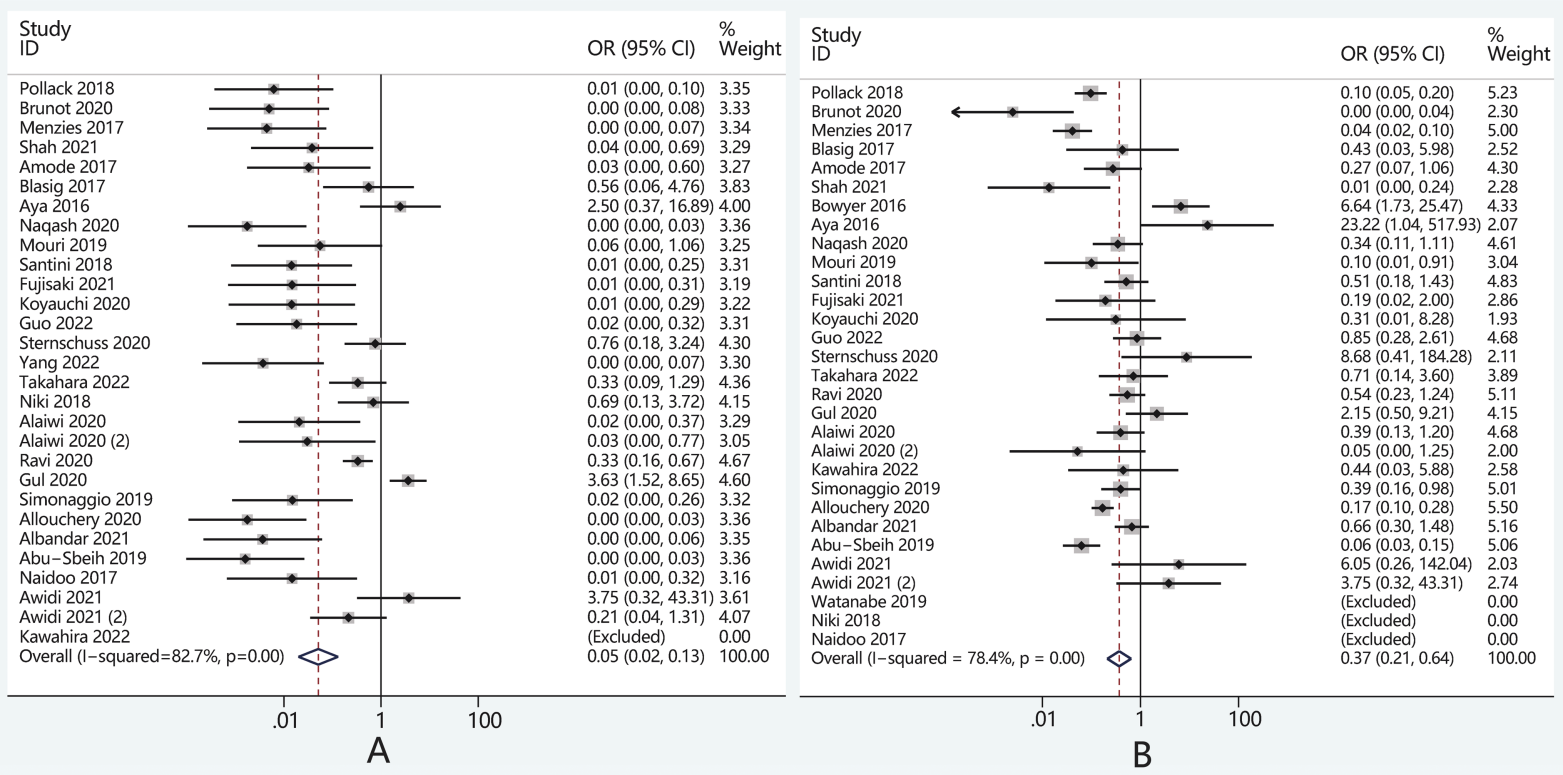
Comparison of the incidence of (A) all-grade and (B) high-grade irAEs in patients after ICI rechallenge versus initial treatment. Abbreviations: ICI, immune checkpoint inhibitors; OR, odds ratio.

Thirty studies were included in the safety analyses. Overall, the incidence of all-grade irAEs and high-grade irAEs caused by rechallenge were 42.6% and 14.5% respectively, lower than that of initial treatment which were 92.5% and 37.3% (Supplementary Table S1). After summarizing the OR of each retrospective study, we found that ICI rechallenge was associated with a significantly lower incidence of all-grade (OR, 0.05; 95%CI 0.02-0.13, *P* < .05) and high-grade irAEs (OR, 0.37; 95%CI 0.21-0.64, *P* < .05) when compared with initial ICI treatment (Figure 2).

**Figure 3.**
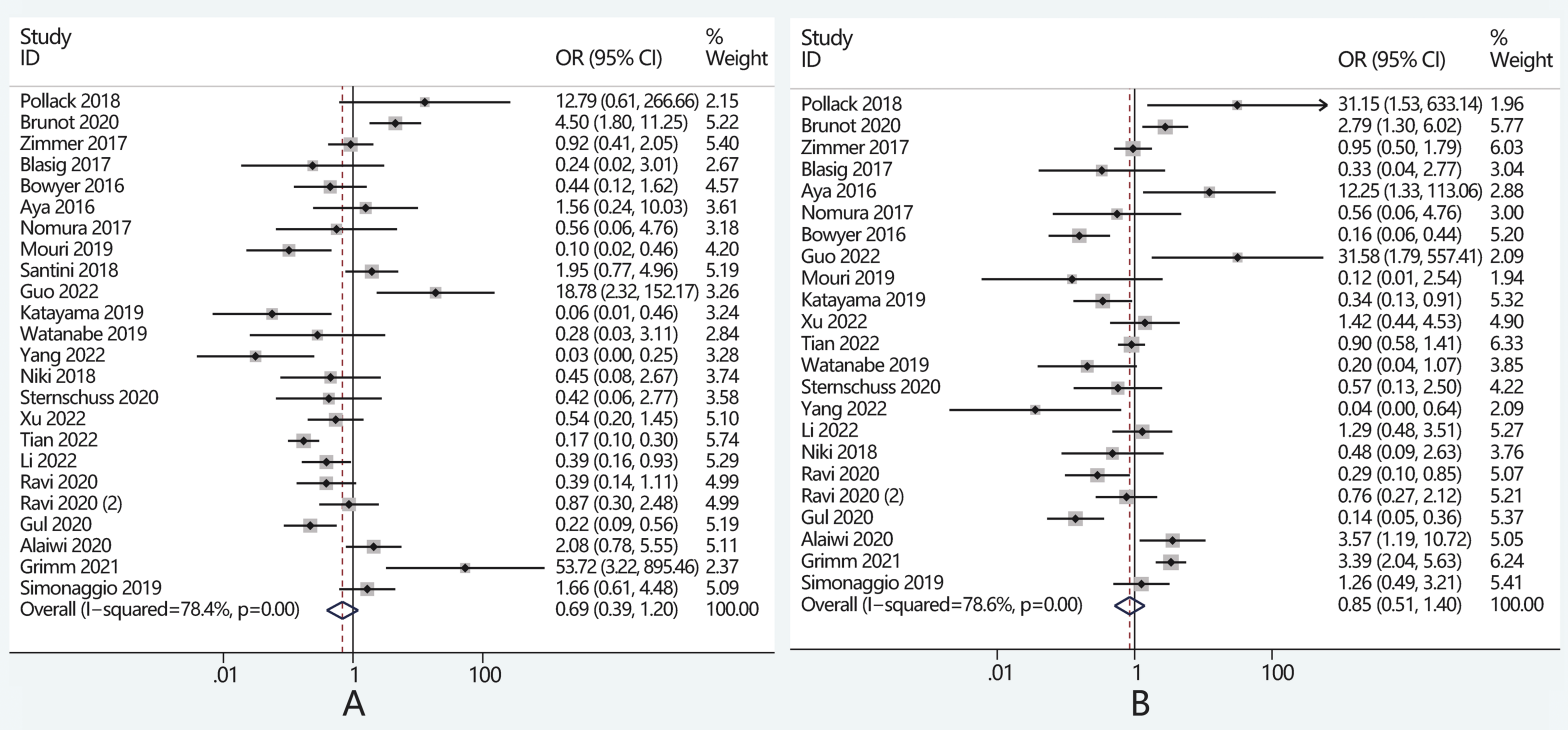
Comparison of (A) ORR and (B) DCR in patients after ICI challenge versus initial treatment. Abbreviations: DCR, disease control rate; ICI, immune checkpoint inhibitors; OR, odds ratio; ORR, objective response rate.

Twenty-four studies reported the efficacy of ICI rechallenge. In the overall cohort, the ORR and DCR of patients receiving ICI rechallenge were 22.9% and 62%, while the ORR and DCR of patients receiving initial immunotherapy were 30.2% and 61.5% (Supplementary Table S1). Further analysis showed no significant difference between the rechallenge and initial ICI treatment group regarding ORR (OR, 0.69; 95%CI, 0.39-1.20, *P* = .29) and DCR (OR, 0.85; 95%CI, 0.51-1.40, *P* = .52) (Figure 3).

### Outcomes of ICI rechallenge when discontinued because of irAEs

**Figure 4.**
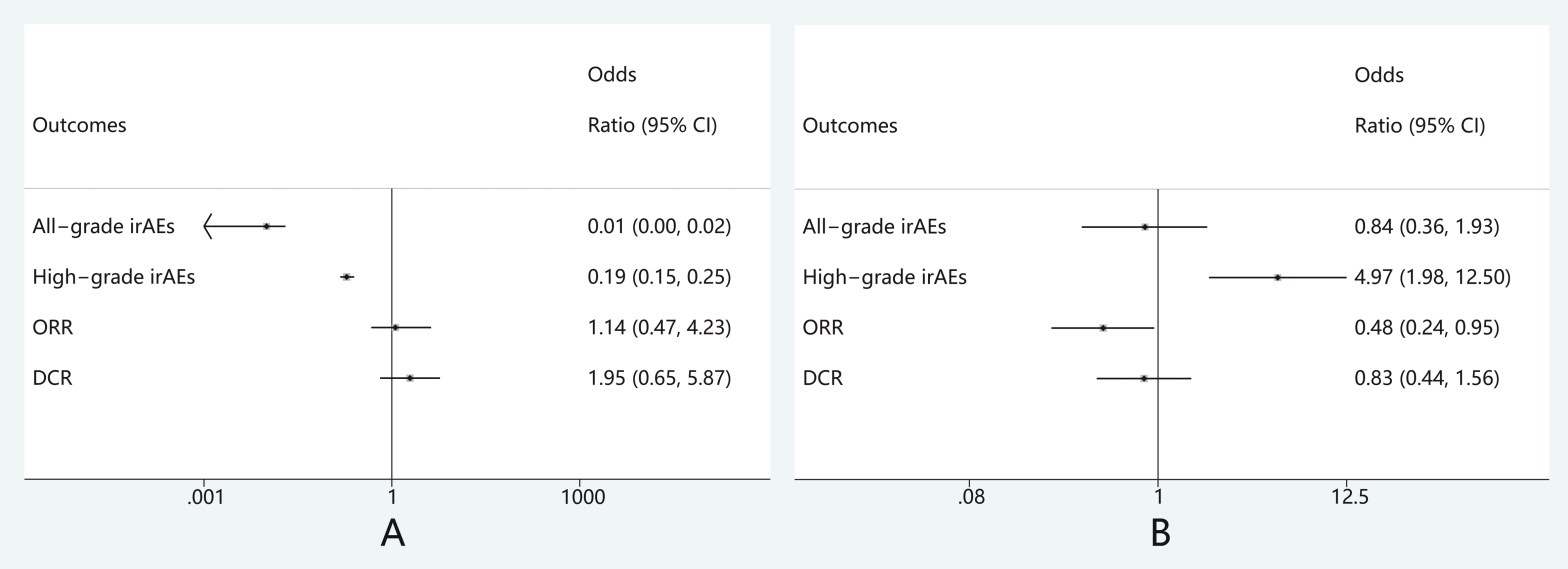
Comparison of outcomes after ICI rechallenge versus initial treatment in patients discontinued initial ICI treatments because of (A) irAEs and (B) PD. Abbreviations: DCR, disease control rate; ICI, immune checkpoint inhibitors; irAEs, immune-related adverse events; OR, odds ratio; ORR, objective response rate; PD, progressive disease.

The results of the meta-regression analysis revealed that the reason for discontinuing initial ICI treatment contributed to the heterogeneity both in the meta-analysis of safety and efficacy. Among patients who stopped initial ICI treatment due to irAEs, the incidence of all-grade and high-grade irAEs were 100% and 43.4% after initial ICI treatment, and were 42.8% and 13.5% after rechallenge treatment (Supplementary Table S1). We found that resuming ICI treatment did not lead to an increased risk of toxicity, as the incidence of both all-grade (OR, 0.01; 95%CI 0.00-0.02, *P* < .05) and high-grade irAEs (OR, 0.19; 95%CI 0.15-0.25, *P* < .05) was significantly decreased (Figure 4).

In the analysis of efficacy, the ORR and DCR were 41.5% and 77.2% for patients receiving ICI rechallenge, and were 34.2% and 65% for patients receiving initial treatment (Supplementary Table S1). Further pooled analysis revealed that for patients who stopped initial ICI treatment owing to irAEs, the ORR and DCR after rechallenge were not significantly different from that of initial treatment (Figure 4).

### Outcomes of ICI rechallenge when discontinued because of PD

Among patients who discontinued their initial ICI treatment due to PD, the incidence of all-grade and high-grade irAEs were 44.4% and 25.9% for patients receiving rechallenge treatment and were 48.1% and 5.6% for patients receiving initial treatment (Supplementary Table S1). The incidence of all-grade irAEs (OR, 0.84; 95%CI 0.36-1.93, *P* = 0.75) caused by rechallenge was not significantly different from that of initial treatment, but there was a significant increase in high-grade irAEs after ICI rechallenge (OR, 4.97; 95%CI, 1.98-12.5, *P* < .05) (Figure 4).

For patients who discontinued ICI treatment due to PD, the ORR and DCR were 25.8% and 57.1% after initial treatment, and were 14.5% and 58.5% after ICI rechallenge (Supplementary Table S1). ICI rechallenge in such patients showed worse efficacy than initial ICI treatment, as the ORR was significantly lower (OR, 0.48; 95%CI, 0.24-0.95, *P* < .05) (Figure 4).

### Safety of ICI rechallenge according to cancer type and rechallenge strategy

Supplementary Table S2 presented rechallenge safety statistics based on cancer type and ICI strategies. In melanoma patients, the incidence of all-grade and high-grade irAEs were 97.5% and 68.6% after initial treatment, and were 48.4% and 25.1% after rechallenge. If rechallenged with the same ICI monotherapy, 62.5% and 12.5% melanoma patients would experience all-grade and high-grade irAEs, respectively. Meanwhile, if rechallenged with a different kind of ICI monotherapy, the incidence of all-grade and high-grade irAEs was 44.7% and 22.9%, respectively. In NSCLC patients, the incidence of all-grade and high-grade irAEs were 92.2% and 14.5% after initial treatment, and were 38.4% and 8.2% after rechallenge. If rechallenged with the same ICI monotherapy, 38.3% and 20% patients with NSCLC would finally experience all-grade and high-grade irAEs. If NSCLC patients were rechallenged with the dual immunotherapy (the same ICI and another kind of ICI), the incidence of all-grade and high-grade irAEs were 40% and 7.6%. In RCC patients, the incidence of all-grade and high-grade irAEs were 66.7% and 25.3% after initial treatment and were 52% and 16% after rechallenge. If patients were rechallenged with dual immunotherapy, the incidence of all-grade and high-grade irAEs were 64.4% and 13.3%, respectively. If rechallenged with the same ICI and targeted therapy, 55.2% and 24.1% RCC patients experienced all-grade and high-grade irAEs.

Further analyses were conducted according to predefined subgroups. As shown in Supplementary Figure S1, patients receiving combined treatments showed similar risk of all-grade (OR, 0.60; 95%CI 0.23-1.55, *P* = .29) and high-grade irAEs (OR, 1.17; 95%CI 0.45-3.04, *P* = .74) after ICI rechallenge. In contrast, the risk of all-grade (OR, 0.02; 95%CI 0.01-0.03, *P* < .05) and high-grade irAEs (OR, 0.23; 95%CI 0.12-0.44, *P* < .05) was dramatically lower in patients who did not receive combined treatments. In patients with RCC, the incidence of all-grade and high-grade irAEs after rechallenge was not significantly different from that of initial treatment. By contrast, patients with other cancer types exhibited lower toxicity after rechallenge when compared to initial treatment. Subgroup analysis showed results consistent with those of the overall population, regardless of whether the same ICI was used in rechallenge.

The efficacy of ICI rechallenge in other subgroups of patients was presented in Supplementary Figure S1.

### Relationship between ICI rechallenge with PFS and OS

**Figure 5.**
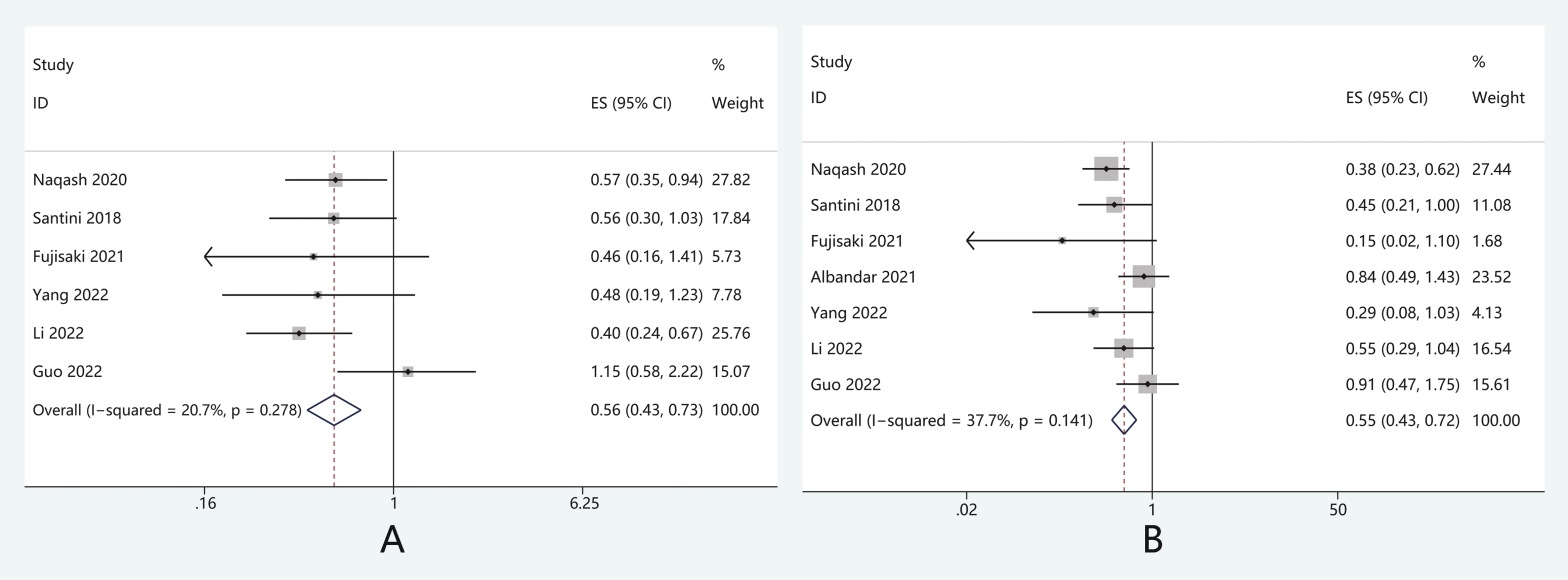
Comparison of (A) PFS and (B) OS in patients after ICI rechallenge versus permanently discontinuation. Abbreviations: HR, hazard ratio; ICI, immune checkpoint inhibitors; OS, overall survival; PFS, progression-free survival.

Of the 39 studies, survival information was available for 7. The pooled HR indicated that the PFS and OS of patients receiving rechallenge were significantly longer than those of patients who permanently discontinued treatment. (PFS: HR, 0.56; 95%CI 0.43-0.73, *P* < .05; OS: HR, 0.55; 95%CI 0.43-0.72, *P* < .05) (Figure 5).

### Publication bias

Funnel plots were constructed to estimate the extent of publication bias in the pooled analyses (Supplemental Figure S2). For the irAEs occurrence rate, ORR and DCR after ICI rechallenge, the funnel plot, and Begg’s test revealed no apparent publication bias among the included comparative studies. We did not test publication bias for the meta-analyses of PFS and OS because too few studies were available to perform a valid statistical test.

## Discussion

To the best of our knowledge, this is the most comprehensive meta-analysis to date. We compiled the available data on the safety and efficacy of ICI rechallenge and, for the first time, evaluated its effect on the long-term survival outcomes of cancer patients. Simultaneously, strict screening was conducted on the baseline characteristics of the included patients to avoid confounding factors as much as possible. We also adopted predefined subgroup analyses and meta-regressions to explore the sources of heterogeneity. Our study revealed that patients with cancer receiving ICI rechallenge did not lead to worse safety than initial ICI therapy, but prolonged survival time.

Notably, the definition of ICI rechallenge remains unclear. It is narrowly defined as recovery of ICI after improvement of irAEs in patients who discontinued treatment due to irAEs.^28^ A more general definition is the resumption of ICI in patients who stopped treatment for any cause, including progressive diseases or adjustment of specific drugs.^29^ Previous studies mainly focused on the narrow definition. However, the general definition may better match complicated real-world clinical practice. Therefore, we included patients discontinued ICI therapy due to irAEs or disease progression in this study.

In the meta-regression analysis, we found that the reason for ceasing initial ICI treatment was the source of heterogeneity and may contribute to the conflicting observations that have emerged so far. In patients who stopped their initial ICI treatment because of irAEs, ICI rechallenge did not lead to more severe toxicity. However, for patients who discontinued ICI therapy because of PD, ICI rechallenge may cause more high-grade irAEs and poorer efficacy. Therefore, it is necessary to distinguish the reasons for stopping initial ICI treatment when designing prospective or other types of studies on this topic in the future, otherwise, the reliability of the conclusion will be weakened.

Theoretically, owing to their non-overlapping mechanisms,^30,31^ switching from anti-PD-(L)1 to anti-CTLA-4 therapy, or vice versa, may be reasonable though the risk of toxicity may increase. In this study, we found that the incidence of all-grade and high-grade irAEs after rechallenge was reduced, regardless of whether the same ICI was used. We also found that using the same ICI for rechallenge was associated with worse efficacy than initial treatment, whereas there was no difference in efficacy for patients using different ICI for rechallenge. However, the cohort shifting from anti-PD-(L)1 to anti-CTLA-4 in this study mainly included patients with melanoma, so caution should be taken for other types of cancer when making the decision to switch to anti-CTLA-4 monotherapy for rechallenge.

According to the latest guidelines, the same type of immunotherapy should usually be discontinued in cases of severe irAEs. Restoring immunotherapy may only be considered following the event of grade 2 or lower irAEs. Our study revealed that when patients stop immunotherapy due to irAEs, rechallenge with the same ICI may be feasible since it did not lead to an increase in the incidence of all-grade and high-grade irAEs. However, because most of the included studies did not provide details of irAEs type, we could not make further analysis based on irAEs type. Though rechallenge has been reported in several cases with previous serious cardiac or neurologic toxicity,^32,33^ it should be considered cautiously and individually especially for patients with serious irAEs involving the neurological or cardiovascular systems. In clinical practice, irAEs involving the neurological or cardiovascular systems are less likely to be rechallenged, whereas those involving the skin and gastrointestinal tract are generally more frequently rechallenged.^14^

In addition, the ASCO guidelines also suggest that if a patient has achieved an objective response to initial ICI treatment, there is a reasonable likelihood that the response will be durable, and resumption of therapy (with attendant risk of recurrence of toxicity) may not be advisable. Conversely, for patients who have not yet responded, resumption of ICI therapy is reasonable. By contrast, we found that the survival of patients receiving ICI rechallenge was significantly prolonged than those who permanently discontinued immunotherapy. These results suggested the potential for the immunotherapy microenvironment “settling down” after several months or years cannot be readily discounted. This possibility could endanger any gains obtained from immunotherapy and lead to disease relapse.^27^ However, it should be noted that the studies we included in our survival analysis mainly focused on NSCLC patients, and our analysis was not only based on patients with objective response but also included patients with SD and PD, which may limit the generalizability of our conclusions.

We also found that ICI rechallenge was associated with worse efficacy in patients receiving combined treatments such as chemotherapy, radiation therapy, or targeted therapy during ICI therapy. One possible reason may be that combination treatments are more commonly used in “cold” tumors. Although ICI has been reported with a high clinical success rate for various types of cancer, numerous patients still bear “cold” tumors with insufficient T-cell infiltration and low immunogenicity, responding poorly to ICI therapy.^34^ Therefore, ICI is usually applied in combination with other therapeutic strategies to turn a “cold” tumor into a “hot” one by modulating the tumor immune microenvironment (TIME), thereby improving the efficiency of immunoncotherapy.^35^ However, these processes involve various molecules and signaling pathways which may simultaneously produce feedback inhibition of anti-tumoral immunity via cross-talk with the PD-1 axis and other key immunosuppressive molecules within the TIME.^36^ For example, inhibition of fibroblast growth factor receptor (FGFR) by Lenvatinib for HCC leads to feedback activation of the EGFR pathway,^37^ which will inhibit antitumor immunity by activating the PD-1/PD-L1 pathway.^38^ Therefore, despite immune activation by combined treatments, after a phase of equilibrium, these “cold” tumors are able to evade immunological control and finally enter in the escape phase characterized by several immunosuppressive traits and independence from any kind of immune control, which may cause the failure of rechallenge. More research is needed to investigate the exact mechanism underlying these observations.

We acknowledge the following limitations of our study: First, this meta-analysis was based on retrospective studies with inherent biases. Second, few studies reported survival outcomes, making it difficult to conduct comprehensive subgroup analyses when pooling the HR for OS and PFS. Finally, because most of the included studies did not provide details of irAEs type, we could not make further analysis based on irAEs type. Therefore, circumspection should be exercised in interpreting these results.

## Conclusion

Our study revealed that ICI rechallenge may be a feasible and effective strategy for patients with cancer with relative safety, similar efficacy, and improved survival outcomes. However, the decision to rechallenge should be weighed carefully and individually, considering the potential benefits and risks. Further large-scale prospective studies with strict and comprehensive inclusion criteria are required to verify our findings.

## Supplementary material

Supplementary material is available at *The Oncologist* online.

oyae134_suppl_Supplementary_Materials

## Data Availability

The datasets that support the findings of this study are available from the corresponding author upon reasonable request.
