# Correction to: Safety, efficacy, and survival outcomes of immune checkpoint inhibitors rechallenge in patients with cancer: a systematic review and meta-analysis

**DOI:** 10.1093/oncolo/oyae224

**Published:** 2024-08-13

This is a correction to: Shi-Jia Liu *et al.*, Safety, efficacy, and survival outcomes of immune checkpoint inhibitors rechallenge in patients with cancer: a systematic review and meta-analysis, *The Oncologist*, 2024; oyae134, https://doi.org/10.1093/oncolo/oyae134

The grant number acknowledging funding from Shandong Provincial Natural Science Foundation has been corrected.

