# Supplementary material for: Safety, efficacy, and survival outcomes of immune checkpoint inhibitors rechallenge in patients with cancer: a systematic review and meta-analysis
Source: Oncologist. 2024 Jun 28;29(11):e1425–34. doi: 10.1093/oncolo/oyae134 (PMC11546642; doi:10.1093/oncolo/oyae134)
Supplement: oyae134_suppl_Supplementary_Materials [file oyae134_suppl_supplementary_materials.zip › oyae134_suppl_Supplementary_Figures_Caption.docx]

**Supplementary Fig. S1. Comparison of (A) incidence of all-grade irAEs, (B) incidence of high-grade irAEs, (C) ORR and (D) DCR** **in patients of the rest subgroups after ICI rechallenge versus initial treatment.** OR, odds ratio; ICI, immune checkpoint inhibitors; ORR, objective response rate; DCR, disease control rate; irAEs, immune-related adverse events; NSCLC, non-small cell lung cancer; RCC, renal cell carcinoma. “Combined treatments” includes surgery, radiotherapy, chemotherapy, or targeted therapy.

**Supplementary Fig. S2. Funnel plots for (A) all-grade irAEs; (B) high-grade irAEs; (C) ORR; (D) DCR.**
