# Supplementary material for: Safety, efficacy, and survival outcomes of immune checkpoint inhibitors rechallenge in patients with cancer: a systematic review and meta-analysis
Source: Oncologist. 2024 Jun 28;29(11):e1425–34. doi: 10.1093/oncolo/oyae134 (PMC11546642; doi:10.1093/oncolo/oyae134)
Supplement: oyae134_suppl_Supplementary_Materials [file oyae134_suppl_supplementary_materials.zip › oyae134_suppl_Supplementary_Table_S1.docx]

| **Table S1.** Characteristics of studies included for safety and efficacy analysis | | | | | | | | | | | | | | | | |
| --- | --- | --- | --- | --- | --- | --- | --- | --- | --- | --- | --- | --- | --- | --- | --- | --- |
| Author | Year | Cancer | Cause of  interruption | Initial treatment | | | | | | Rechallenge | | | | | | Combined treatments |
|  |  |  |  | ICI type | All-grade irAEs | High-grade irAEs | ORR | DCR | mPFS | ICI type | All-grade irAEs | High-grade irAEs | ORR | DCR | mPFS |  |
| Pollack^1^ | 2018 | Melanoma | irAEs | Dual | 80/80 | 55/80 | 0/13 | 0/13 |  | PD-(L)1 | 40/80 | 14/80 | 4/13 | 7/13 |  | No |
| Brunot^2^ | 2020 | Melanoma | irAEs | CTLA-4 | 56/56 | 56/56 | 8/56 | 23/56 |  | PD-(L)1 | 56/56 | 12/56 | 24/56 | 37/56 |  | No |
| Zimmer^3^ | 2017 | Melanoma | irAEs | PD-(L)1 | NA | NA | 15/84 | 30/84 |  | Dual | NA | NA | 14/84 | 29/84 | 3 (2.8-3.8) * | No |
| Menzies^4^ | 2017 | Melanoma | irAEs | CTLA-4 | 67/67 | 58/67 | NA | NA |  | PD-(L)1 | 58/67 | 14/67 | NA | NA | 7.2 (3.1-11.3) * | No |
| Shah^5^ | 2021 | Melanoma | irAEs | Dual | 32/32 | 32/32 | NA | NA |  | Dual | 32/32 | 15/32 | 25/32 | 25/32 |  | No |
| Blasig^6^ | 2017 | Melanoma | PD | PD-(L)1 | 6/8 | 2/8 | 3/8 | 6/8 |  | PD-(L)1 | 2/8 | 1/8 | 1/8 | 4/8 |  | Yes |
| Bowyer^7^ | 2016 | Melanoma | PD | PD-(L)1 | NA | 3/40 | 8/40 | 23/40 |  | CTLA-4 | NA | 14/40 | 4/40 | 7/40 |  | No |
| Aya^8^ | 2016 | Melanoma | PD | PD-(L)1 | 4/9 | 0/9 | 4/9 | 2/9 |  | CTLA-4 | 6/9 | 5/9 | 5/9 | 7/9 | 3.1 (2.6-3.7) * | Yes |
| Nomura^9^ | 2017 | Melanoma | irAEs /PD | PD-(L)1 | NA | NA | 3/8 | 6/8 | 4.1 (range 2.1-8.4) | PD-(L)1 | NA | NA | 2/8 | 5/8 | 4.3 (range 0.3-14) | Yes |
| Amode^10^ | 2017 | Melanoma | irAEs | CTLA-4 | 23/23 | 10/23 | NA | NA |  | PD-(L)1 | 14/23 | 4/23 | NA | NA |  | No |
| Mouri^11^ | 2019 | NSCLC | irAEs | PD-(L)1 | 21/21 | 7/21 | 13/21 | 21/21 |  | PD-(L)1 | 15/21 | 1/21 | 3/21 | 18/21 |  | No |
| Santini^12^ | 2018 | NSCLC | irAEs | PD-(L)1 | 38/38 | 13/38 | 12/38 | NA |  | PD-(L)1 | 20/38 | 8/38 | 18/38 | NA |  | No |
| Fujisaki^13^ | 2021 | NSCLC | irAEs | PD-(L)1 | 14/14 | 4/14 | NA | NA |  | PD-(L)1 | 4/14 | 1/14 | NA | NA |  | No |
| Koyauchi^14^ | 2020 | NSCLC | irAEs | PD-(L)1 | 16/16 | 1/16 | NA | NA |  | PD-(L)1 | 5/16 | 0/16 | 8/16 | 14/16 |  | No |
| Guo^15^ | 2022 | NSCLC | irAEs | PD-(L)1 | 40/40 | 8/40 | 27/40 | 29/40 |  | PD-(L)1 | 24/40 | 7/40 | 39/40 | 40/40 |  | No |
| Katayama^16^ | 2019 | NSCLC | PD | PD-(L)1 | NA | NA | 12/35 | 24/35 | 4 (2.8–4.6) * | PD-(L)1 | NA | NA | 1/35 | 15/35 | 2.7 (1.4–3.7) * | No |
| Xu^17^ | 2022 | NSCLC | PD | PD-(L)1 | NA | NA | 14/40 | 32/40 | 5.7 (4.1-7.2) * | PD-(L)1 | NA | NA | 9/40 | 34/40 | 6.8 (5.8–7.8) * | Yes |
| Tian^18^ | 2022 | NSCLC | PD | PD-(L)1 | NA | NA | 76/204 | 155/204 | 5.3 | PD-(L)1 | NA | NA | 19/204 | 152/204 | 5.0 (4.5-5.5) * | Yes |
| Naqash^19^ | 2020 | NSCLC | irAEs | PD-(L)1 | 138/138 | 11/138 | NA | NA |  | PD-(L)1 | 45/138 | 4/138 | NA | NA |  | No |
| Watanabe^20^ | 2019 | NSCLC | PD | PD-(L)1 | NA | 0/14 | 3/14 | 8/14 | 3.7 (1.3-7.1) * | PD-(L)1 | NA | 0/14 | 1/14 | 3/14 | 1.6 (0.8-2.6) * | Yes |
| Sternschuss^21^ | 2020 | NSCLC | PD | PD-(L)1 | 7/15 | 0/15 | 4/15 | 7/15 |  | Dual | 6/15 | 3/15 | 2/15 | 5/15 |  | Yes |
| Li^22^ | 2022 | NSCLC | PD | PD-(L)1 | NA | NA | 23/45 | 34/45 |  | PD-(L)1 | NA | NA | 13/45 | 36/45 |  | Yes |
| Yang^23^ | 2022 | NSCLC | irAEs /PD | PD-(L)1 | 40/40 | NA | 18/40 | 40/40 |  | PD-(L)1 | 9/40 | 1/40 | 1/40 | 30/40 |  | No |
| Takahara^24^ | 2022 | NSCLC | irAEs /PD | PD-(L)1 | 9/24 | 4/24 | NA | NA |  | PD-(L)1 | 4/24 | 3/24 | NA | NA |  | No |
| Niki^25^ | 2018 | NSCLC | irAEs /PD | PD-(L)1 | 6/11 | 0/11 | 5/11 | 7/11 | 4.9 | PD-(L)1 | 5/11 | 0/11 | 3/11 | 5/11 | 2.7 | Yes |
| Ravi^26^ | 2020 | RCC | irAEs /PD | PD-(L)1 | 49/69 | 18/69 | 14/38 | 32/38 |  | PD-(L)1 | 31/69 | 11/69 | 7/38 | 23/38 |  | Yes |
| Ravi (2) | 2020 | RCC | irAEs /PD | Dual |  |  | 11/31 | 20/31 |  | Dual |  |  | 10/31 | 18/31 |  | Yes |
| Gul^27^ | 2020 | RCC | irAEs /PD | PD-(L)1 | 15/45 | 3/45 | 24/45 | 36/45 |  | Dual | 29/45 | 6/45 | 9/45 | 16/45 | 4 (range 0.8-19) | Yes |
| Alaiwi^28^ | 2020 | RCC | irAEs | PD-(L)1 | 29/29 | 13/29 | 10/36 | 16/36 |  | PD-(L)1 | 16/29 | 7/29 | 21/36 | 30/36 |  | Yes |
| Alaiwi (2) | 2020 | RCC | irAEs | Dual | 7/7 | 4/7 |  |  |  | PD-(L)1 | 2/7 | 0/7 |  |  |  | Yes |
| Grimm^29^ | 2021 | RCC | PD | PD-(L)1 | NA | NA | 0/135 | 38/135 |  | Dual | NA | NA | 22/135 | 77/135 |  | No |
| Kawahira^30^ | 2022 | Multiple | irAEs | Dual | 10/10 | 2/10 | NA | NA |  | Dual | 10/10 | 1/10 | NA | NA |  | No |
| Simonaggio^31^ | 2019 | Multiple | irAEs | PD-(L)1 | 40/40 | 22/40 | 9/40 | 26/40 |  | PD-(L)1 | 22/40 | 13/40 | 13/40 | 28/40 |  | No |
| Allouchery^32^ | 2019 | Multiple | irAEs | Dual | 180/180 | 88/180 | NA | NA |  | Dual | 70/180 | 25/180 | NA | NA |  | No |
| Albandar^33^ | 2021 | Multiple | irAEs | Dual | 98/98 | 17/98 | NA | NA |  | Dual | 41/98 | 12/98 | NA | NA |  | No |
| Abu-Sbeih^34^ | 2019 | Multiple | irAEs | Dual | 165/165 | 62/165 | NA | NA |  | Dual | 57/165 | 6/165 | NA | NA |  | No |
| Naidoo^35^ | 2017 | Multiple | irAEs | PD-(L)1 | 12/12 | 0/12 | NA | NA |  | PD-(L)1 | 3/12 | 0/12 | NA | NA |  | No |
| Awidi^36^ | 2021 | Multiple | PD | PD-(L)1 | 1/11 | 0/11 | NA | NA |  | PD-(L)1 | 3/11 | 2/11 | NA | NA |  | Yes |
| Awidi (2) | 2021 | Multiple | PD | Dual | 8/11 | 1/11 | NA | NA |  | Dual | 4/11 | 3/11 | NA | NA |  | Yes |
|  |  |  | irAEs |  | 100% | 43.4% | 34.2% | 65% |  |  | 42.8% | 13.5% | 41.5% | 77.2% |  |  |
|  |  |  | PD |  | 48.1% | 5.6% | 25.8% | 57.1% |  |  | 44.4% | 25.9% | 14.5% | 58.5% |  |  |
| Overall cohort | | | | | 92.5% | 37.3% | 30.2% | 61.5% |  |  | 42.6% | 14.5% | 22.9% | 62% |  |  |
| *mPFS are presented as month and 95%CI.  NSCLC, non-small cell lung cancer; RCC, renal cell carcinoma; NA, not applicable; PD-1, programmed cell death protein 1; PD-L1, programmed cell death ligand 1; CTLA-4, cytotoxic T lymphocyte antigen 4; irAEs, immune-related adverse events; PD, progressive disease; ORR, objective responses rate; DCR, disease control rate; ICI, immune checkpoint inhibitors; mPFS, median progression-free survival.  “Multiple” means the cohort enrolled more than one kind of cancer patients; “Dual” means PD-(L)1 and CTLA-4; “Combined treatments” includes surgery, radiotherapy, chemotherapy, or targeted therapy. | | | | | | | | | | | | | | | | |

**References**

1. Pollack MH, Betof A, Dearden H, et al. Safety of resuming anti-PD-1 in patients with immune-related adverse events (irAEs) during combined anti-CTLA-4 and anti-PD1 in metastatic melanoma. *Ann Oncol*. Jan 1 2018;29(1):250-255. doi:10.1093/annonc/mdx642

2. Brunot A, Grob JJ, Jeudy G, et al. Association of Anti-Programmed Cell Death 1 Antibody Treatment With Risk of Recurrence of Toxic Effects After Immune-Related Adverse Events of Ipilimumab in Patients With Metastatic Melanoma. *JAMA Dermatol*. Sep 1 2020;156(9):982-986. doi:10.1001/jamadermatol.2020.2149

3. Zimmer L, Apuri S, Eroglu Z, et al. Ipilimumab alone or in combination with nivolumab after progression on anti-PD-1 therapy in advanced melanoma. *Eur J Cancer*. Apr 2017;75:47-55. doi:10.1016/j.ejca.2017.01.009

4. Menzies AM, Johnson DB, Ramanujam S, et al. Anti-PD-1 therapy in patients with advanced melanoma and preexisting autoimmune disorders or major toxicity with ipilimumab. *Ann Oncol*. Feb 1 2017;28(2):368-376. doi:10.1093/annonc/mdw443

5. Shah P, Punekar SR, Pavlick AC. Response to immune checkpoint inhibitor rechallenge after high-grade immune related adverse events in patients with advanced melanoma. *Melanoma Res*. Jun 1 2021;31(3):242-248. doi:10.1097/cmr.0000000000000730

6. Blasig H, Bender C, Hassel JC, et al. Reinduction of PD1-inhibitor therapy: first experience in eight patients with metastatic melanoma. *Melanoma Res*. Aug 2017;27(4):321-325. doi:10.1097/cmr.0000000000000341

7. Bowyer S, Prithviraj P, Lorigan P, et al. Efficacy and toxicity of treatment with the anti-CTLA-4 antibody ipilimumab in patients with metastatic melanoma after prior anti-PD-1 therapy. *Br J Cancer*. May 10 2016;114(10):1084-9. doi:10.1038/bjc.2016.107

8. Aya F, Gaba L, Victoria I, et al. Ipilimumab after progression on anti-PD-1 treatment in advanced melanoma. *Future Oncol*. Dec 2016;12(23):2683-2688. doi:10.2217/fon-2016-0037

9. Nomura M, Otsuka A, Kondo T, et al. Efficacy and safety of retreatment with nivolumab in metastatic melanoma patients previously treated with nivolumab. *Cancer Chemother Pharmacol*. Nov 2017;80(5):999-1004. doi:10.1007/s00280-017-3444-0

10. Amode R, Baroudjian B, Kowal A, et al. Anti-programmed cell death protein 1 tolerance and efficacy after ipilimumab immunotherapy: observational study of 39 patients. *Melanoma Res*. Apr 2017;27(2):110-115. doi:10.1097/cmr.0000000000000313

11. Mouri A, Kaira K, Yamaguchi O, et al. Clinical difference between discontinuation and retreatment with nivolumab after immune-related adverse events in patients with lung cancer. *Cancer Chemother Pharmacol*. Oct 2019;84(4):873-880. doi:10.1007/s00280-019-03926-y

12. Santini FC, Rizvi H, Plodkowski AJ, et al. Safety and Efficacy of Re-treating with Immunotherapy after Immune-Related Adverse Events in Patients with NSCLC. *Cancer Immunol Res*. Sep 2018;6(9):1093-1099. doi:10.1158/2326-6066.Cir-17-0755

13. Fujisaki T, Watanabe S, Ota T, et al. The Prognostic Significance of the Continuous Administration of Anti-PD-1 Antibody via Continuation or Rechallenge After the Occurrence of Immune-Related Adverse Events. *Front Oncol*. 2021;11:704475. doi:10.3389/fonc.2021.704475

14. Koyauchi T, Inui N, Karayama M, et al. Clinical Outcomes of Anti-programmed Death-1 Antibody–Related Pneumonitis in Patients with Non-Small Cell Lung Cancer. *SN Comprehensive Clinical Medicine*. 2020/05/01 2020;2(5):570-578. doi:10.1007/s42399-020-00259-3

15. Guo M, VanderWalde AM, Yu X, Vidal GA, Tian GG. Immune Checkpoint Inhibitor Rechallenge Safety and Efficacy in Stage IV Non-Small Cell Lung Cancer Patients After Immune-Related Adverse Events. *Clin Lung Cancer*. Dec 2022;23(8):686-693. doi:10.1016/j.cllc.2022.07.015

16. Katayama Y, Shimamoto T, Yamada T, et al. Retrospective Efficacy Analysis of Immune Checkpoint Inhibitor Rechallenge in Patients with Non-Small Cell Lung Cancer. *J Clin Med*. Dec 31 2019;9(1)doi:10.3390/jcm9010102

17. Xu Z, Hao X, Yang K, et al. Immune checkpoint inhibitor rechallenge in advanced or metastatic non-small cell lung cancer: a retrospective cohort study. *J Cancer Res Clin Oncol*. Nov 2022;148(11):3081-3089. doi:10.1007/s00432-021-03901-2

18. Tian T, Yu M, Yu Y, et al. Immune checkpoint inhibitor (ICI)-based treatment beyond progression with prior immunotherapy in patients with stage IV non-small cell lung cancer: a retrospective study. *Transl Lung Cancer Res*. Jun 2022;11(6):1027-1037. doi:10.21037/tlcr-22-376

19. Naqash AR, Ricciuti B, Owen DH, et al. Outcomes associated with immune-related adverse events in metastatic non-small cell lung cancer treated with nivolumab: a pooled exploratory analysis from a global cohort. *Cancer Immunol Immunother*. Jul 2020;69(7):1177-1187. doi:10.1007/s00262-020-02536-5

20. Watanabe H, Kubo T, Ninomiya K, et al. The effect and safety of immune checkpoint inhibitor rechallenge in non-small cell lung cancer. *Jpn J Clin Oncol*. Aug 1 2019;49(8):762-765. doi:10.1093/jjco/hyz066

21. Sternschuss M, Peled N, Allen AM, et al. Can Ipilimumab restore immune response in advanced NSCLC after progression on anti-PD-1/PD-L1 agents? *Thorac Cancer*. Aug 2020;11(8):2331-2334. doi:10.1111/1759-7714.13502

22. Li L, Liu T, Liu Q, et al. Rechallenge of immunotherapy beyond progression in patients with extensive-stage small-cell lung cancer. *Front Pharmacol*. 2022;13:967559. doi:10.3389/fphar.2022.967559

23. Yang J, Zeng R, Zhou J, et al. Efficacy, prognosis and safety analysis of anti-PD-1/PD-L1 inhibitor rechallenge in advanced lung cancer patients: a cohort study. *Transl Lung Cancer Res*. Jun 2022;11(6):1038-1050. doi:10.21037/tlcr-22-360

24. Takahara Y, Tanaka T, Ishige Y, et al. Efficacy and predictors of rechallenge with immune checkpoint inhibitors in non-small cell lung cancer. *Thorac Cancer*. Feb 2022;13(4):624-630. doi:10.1111/1759-7714.14309

25. Niki M, Nakaya A, Kurata T, et al. Immune checkpoint inhibitor re-challenge in patients with advanced non-small cell lung cancer. *Oncotarget*. Aug 17 2018;9(64):32298-32304. doi:10.18632/oncotarget.25949

26. Ravi P, Mantia C, Su C, et al. Evaluation of the Safety and Efficacy of Immunotherapy Rechallenge in Patients With Renal Cell Carcinoma. *JAMA Oncol*. Oct 1 2020;6(10):1606-1610. doi:10.1001/jamaoncol.2020.2169

27. Gul A, Stewart TF, Mantia CM, et al. Salvage Ipilimumab and Nivolumab in Patients With Metastatic Renal Cell Carcinoma After Prior Immune Checkpoint Inhibitors. *J Clin Oncol*. Sep 20 2020;38(27):3088-3094. doi:10.1200/jco.19.03315

28. Abou Alaiwi S, Xie W, Nassar AH, et al. Safety and efficacy of restarting immune checkpoint inhibitors after clinically significant immune-related adverse events in metastatic renal cell carcinoma. *J Immunother Cancer*. Feb 2020;8(1)doi:10.1136/jitc-2019-000144

29. Grimm M-O, Esteban E, Barthélémy P, et al. Efficacy of nivolumab/ipilimumab in patients with initial or late progression with nivolumab: Updated analysis of a tailored approach in advanced renal cell carcinoma (TITAN-RCC). *Journal of Clinical Oncology*. 2021/05/20 2021;39(15_suppl):4576-4576. doi:10.1200/JCO.2021.39.15_suppl.4576

30. Kawahira M, Kanmura S, Mizuno K, et al. Effects of immune checkpoint inhibitor therapy resumption in patients with malignant tumors after moderate-to-severe immune-related adverse events. *PLoS One*. 2022;17(4):e0267572. doi:10.1371/journal.pone.0267572

31. Simonaggio A, Michot JM, Voisin AL, et al. Evaluation of Readministration of Immune Checkpoint Inhibitors After Immune-Related Adverse Events in Patients With Cancer. *JAMA Oncol*. Sep 1 2019;5(9):1310-1317. doi:10.1001/jamaoncol.2019.1022

32. Allouchery M, Lombard T, Martin M, et al. Safety of immune checkpoint inhibitor rechallenge after discontinuation for grade ≥2 immune-related adverse events in patients with cancer. *J Immunother Cancer*. Dec 2020;8(2)doi:10.1136/jitc-2020-001622

33. Albandar HJ, Fuqua J, Albandar JM, Safi S, Merrill SA, Ma PC. Immune-Related Adverse Events (irAE) in Cancer Immune Checkpoint Inhibitors (ICI) and Survival Outcomes Correlation: To Rechallenge or Not? *Cancers (Basel)*. Feb 27 2021;13(5)doi:10.3390/cancers13050989

34. Abu-Sbeih H, Ali FS, Naqash AR, et al. Resumption of Immune Checkpoint Inhibitor Therapy After Immune-Mediated Colitis. *J Clin Oncol*. Oct 20 2019;37(30):2738-2745. doi:10.1200/jco.19.00320

35. Naidoo J, Wang X, Woo KM, et al. Pneumonitis in Patients Treated With Anti-Programmed Death-1/Programmed Death Ligand 1 Therapy. *J Clin Oncol*. Mar 2017;35(7):709-717. doi:10.1200/jco.2016.68.2005

36. Awidi M, Connell B, Johnson D, et al. Safety of sequential immune checkpoint inhibitors after prior immune therapy. *J Cancer Res Clin Oncol*. Jun 21 2022;doi:10.1007/s00432-022-04137-4
