# Supplementary material for: Safety, efficacy, and survival outcomes of immune checkpoint inhibitors rechallenge in patients with cancer: a systematic review and meta-analysis
Source: Oncologist. 2024 Jun 28;29(11):e1425–34. doi: 10.1093/oncolo/oyae134 (PMC11546642; doi:10.1093/oncolo/oyae134)
Supplement: oyae134_suppl_Supplementary_Materials [file oyae134_suppl_supplementary_materials.zip › oyae134_suppl_Supplementary_Table_S2.docx]

| **Table S2.** Safety of rechallenge based on different ICI strategies | | | | | | | |
| --- | --- | --- | --- | --- | --- | --- | --- |
| Cancer | Rechallenge ICI strategies | All-grade irAEs rate | | | High-grade irAEs rate | | |
|  |  | initial | rechallenge | OR* | initial | rechallenge | OR* |
| Melanoma | Overall cohort | 97.5% | 48.4% | 0.05 (0.01-0.38) | 68.6% | 25.1% | 0.20 (0.04-0.98) |
|  | Same ICI monotherapy as initial treatment | 75% | 62.5% | 0.56 (0.07-4.76) | 25% | 12.5% | 0.43 (0.03-5.99) |
|  | Different ICI monotherapy | 97.9% | 44.7% | 0.03 (0.00-0.49) | 66.2% | 22.9% | 0.26 (0.04-1.65) |
| NSCLC | Overall cohort | 92.2% | 38.4% | 0.05 (0.01-0.19) | 14.5% | 8.2% | 0.51 (0.30-0.87) |
|  | Same ICI monotherapy as initial treatment | 94.2% | 38.3% | 0.03 (0.01-0.14) | 0 | 20% | 8.68 (0.41-184.28) |
|  | Same ICI as a backbone combined with another type of ICI | 46.7% | 40% | 0.76 (0.18-3.24) | 15.2% | 7.6% | 0.47 (0.27-0.80) |
| RCC | Overall cohort | 66.7% | 52% | 0.26 (0.03-2.01) | 25.3% | 16% | 0.57 (0.31-1.04) |
|  | Same ICI** as a backbone combined with targeted therapy | 100% | 55.2% | 0.02 (0.00-0.37) | 44.8% | 24.1% | 0.39 (0.13-1.20) |
|  | Same ICI as a backbone combined with another type of ICI | 33.3% | 64.4% | 3.63 (1.52-8.65) | 6.7% | 13.3% | 2.15 (0.50-9.21) |
| *Data are presented as OR and 95%CI.  **Including ICI monotherapy or dual immunotherapy of two ICI types.  NSCLC, non-small cell lung cancer; RCC, renal cell carcinoma; ICI, immune checkpoint inhibitors; irAEs, immune-related adverse events | | | | | | | |
