# Supplementary figures and images for: Safety, efficacy, and survival outcomes of immune checkpoint inhibitors rechallenge in patients with cancer: a systematic review and meta-analysis
Source: Oncologist. 2024 Jun 28;29(11):e1425–34. doi: 10.1093/oncolo/oyae134 (PMC11546642; doi:10.1093/oncolo/oyae134)

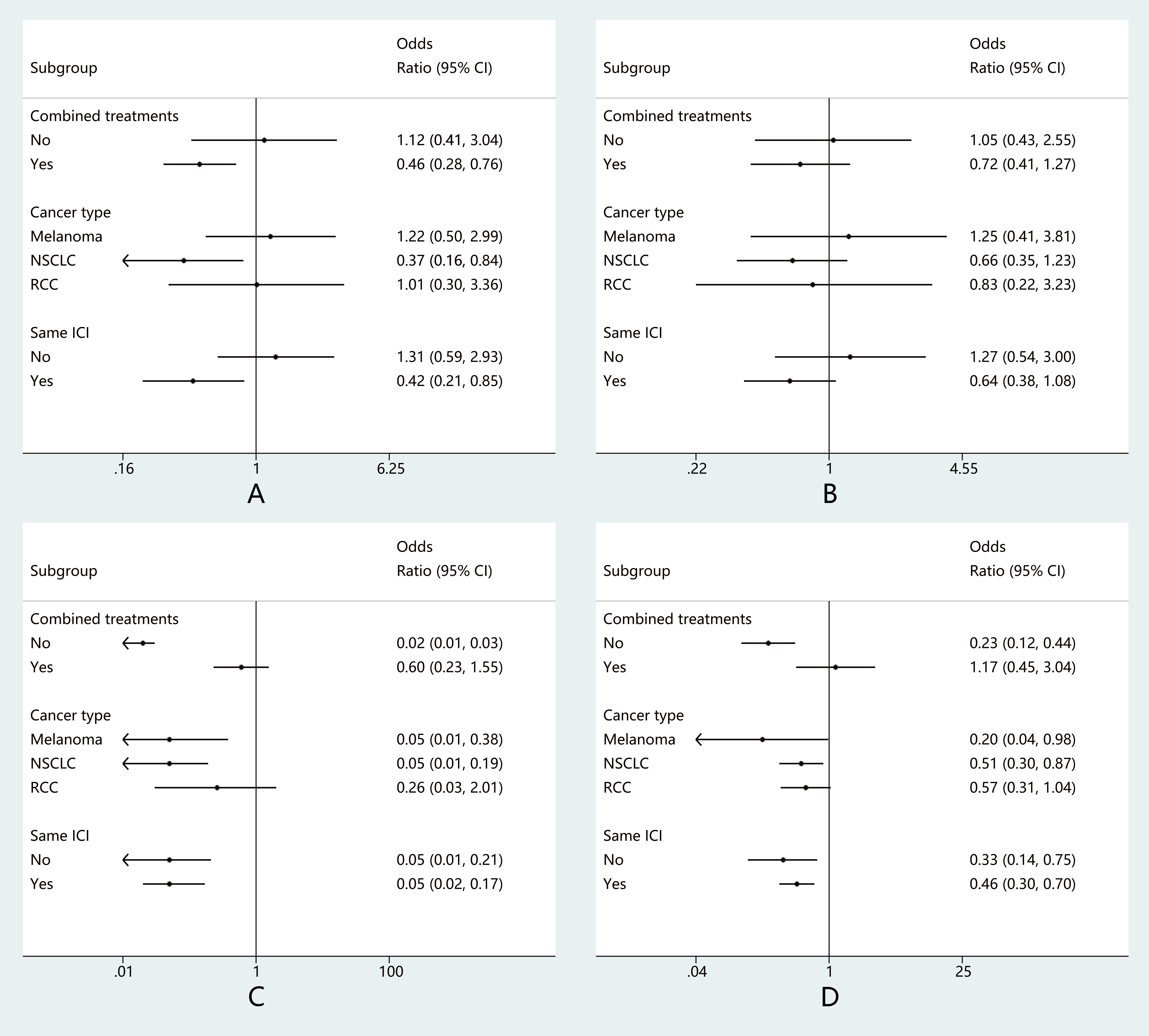

Supplement: oyae134_suppl_Supplementary_Materials [file oyae134_suppl_supplementary_materials.zip › oyae134_suppl_Supplementary_Figure_S1.tif]

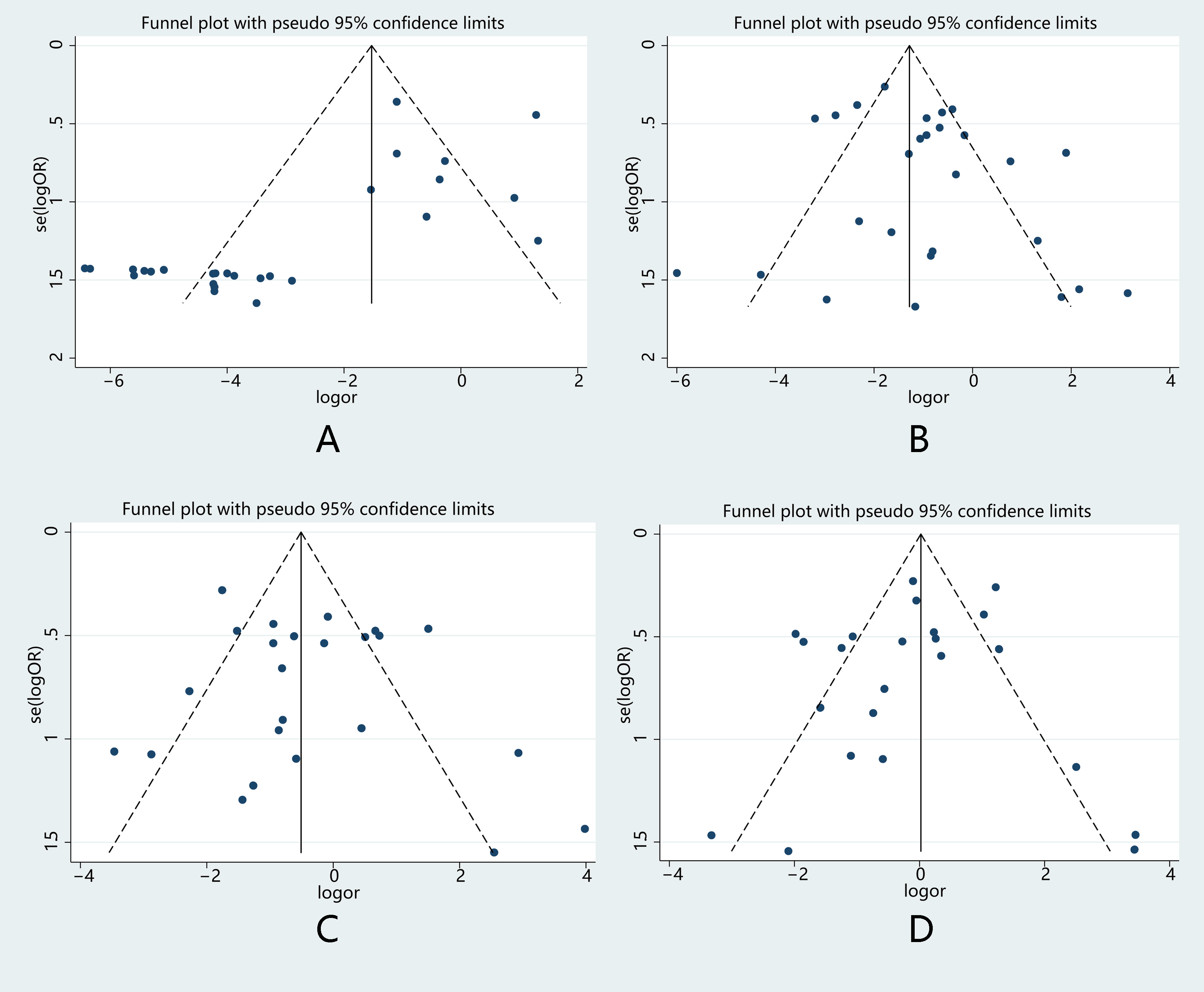

Supplement: oyae134_suppl_Supplementary_Materials [file oyae134_suppl_supplementary_materials.zip › oyae134_suppl_Supplementary_Figure_S2.tif]
